# Ribosomal protein L34 promotes the proliferation, invasion and metastasis of pancreatic cancer cells

**DOI:** 10.18632/oncotarget.13269

**Published:** 2016-11-10

**Authors:** Feng Wei, Lijuan Ding, Zhentong Wei, Yandong Zhang, Yang Li, Luo Qinghua, Yuteng Ma, Liang Guo, Guoyue Lv, Yan Liu

**Affiliations:** ^1^ Department of Hepatobiliary & Pancreas Surgery, The First Hospital of Jilin University, Changchun, Jilin 130021, P.R. China; ^2^ Genetic Engineering Laboratory of PLA, The Eleventh Institute of Academy of Military Medical Sciences of PLA, Jilin 130122, P.R. China; ^3^ Department of Pathophysiology, Basic College of Medicine, Jilin University, Changchun, Jilin 130021, P.R. China; ^4^ Oncologic Gynecology, The First Hospital of Jilin University, Changchun, Jilin 130021, P.R. China; ^5^ Pathology, The First Hospital of Jilin University, Changchun, Jilin 130021, P.R. China

**Keywords:** RPL34, pancreatic cancer, MAPK, metastasis, ingenuity pathway analysis

## Abstract

Ribosomal proteins (RPs) are the main components of ribosomes and participate in the self-assembly of ribosomes and protein synthesis. Recent advance has shown that RPs play important roles in the tumorigenesis and drug resistance of various cancers. However, the expression status and function of RPL34 in pancreatic cancer (PC) remains unclear. In this study, we find that RPL34 is overexpressed in PC tissues and cell lines, which is correlated with decreased methylation of its promoter. Knockdown of RPL34 effectively suppresses the proliferation, colony formation, migration and drug-resistance of PC cells, which are accompanied by cell cycle arrest at the G2 phase and induction of apoptosis. *In vivo* assays demonstrate that RPL34 silencing inhibits PC tumor growth and metastasis. Moreover, gene expression profiling revealed that RPL34 silencing results in alteration of the MAPK and p53 signaling pathways. Clinically, our data indicate a positive association of RPL34 expression with tumor stage and metastasis in PCs. We revealed that RPL34 acts as a potential onco-protein in PC, and RPL34 may be a promising biomarker for prognosis prediction and a potential target for the treatment of PC.

## INTRODUCTION

Pancreatic cancer (PC) is a leading cause of cancer mortality worldwide with over 330,000 new cases and approximately the same number of deaths annually [1]. Moreover, PC may become the second cause of cancer death due to the mortality of PC deaths is likely to further rise in the coming years in the United States (US) [2]. Over the past two decades, great advances have been made in treatments for PC, including surgical resection, chemotherapy, and radiotherapy, both individually and in combination, however, the prognosis for PC patients, particularly those with advanced PC, remains very poor, with a 5-year survival rate of less than 5%. Characterizing the molecular mechanisms underlying PC tumorigenesis may aid development of personalized treatments and identify biomarkers for more effective diagnosis and therapy of PC.

Ribosomal proteins (RPs) are components of ribosomes involved in protein translation and ribosome assembly [3], which are required for the growth and survival of all types of cells [4]. Dysregulation of RPs is associated with various pathological conditions including tumorigenesis. S6K1 regulates hematopoietic stem cell self-renewal and leukemogenesis, while RPL27A contributes to the myelodysplastic phenotype through ribosomal dysgenesis [5, 6]. Elevated RPL6 and RPS13 promote cell proliferation and cell cycle progression in gastric cancer cells [7, 8]. Moreover, phosphorylation of RPS6 attenuates DNA damage and tumor suppression in PC [9], and upregulation of RPS3 and RPL23 promotes drug resistance in gastric cancer cells [10]. Notably, RPL11, RPL5, RPS14, RPL4 and RPS7 could stabilize p53 protein and function as the modulators of p53-MDM2 interaction [11, 12]. These findings suggest that RPs may represent potential biomarkers for cancer diagnosis and characterization of the role of RPs may aid the development of molecular targeted cancers therapies.

RPL34 belongs to the ribosome 60S large subunit and contains a zinc finger motif. In addition to functioning as a ribosomal protein, RPL34 has been reported to play an important role in other cellular processes. RPL34 regulates cell cycle transition by inhibiting Cdk4 and Cdk5 in HeLa cells [13], and over-expression of RPL34 promotes cell proliferation in human non-small cell lung cancer and gastric cancer [14, 15]. However, the biological function and clinical significance of RPL34 in human PC remains largely unknown.

In this study, we found that RPL34 was over-expressed in human PC tissues and cells, and decreased methylation of its promoter region might contribute to upregulation of RPL34. Further, knockdown of RPL34 by lentivirus-delivered siRNA suppressed the proliferation, growth, invasion and migration of PC cells *in vitro* and *in vivo*. In addition, microarray and IPA analyses revealed that RPL34 might work as an oncoprotein in PCs by regulating mitogen-activated protein kinase (MAPK) and p53 signal networks. Our findings reveal a potential oncogenic role for RPL34 and recommend it as a new target for the diagnosis and therapy of PCs.

## RESULTS

### RPL34 is up-regulated in pancreatic cancer and correlated with a poor prognosis of PC patients

**Figure 1:**
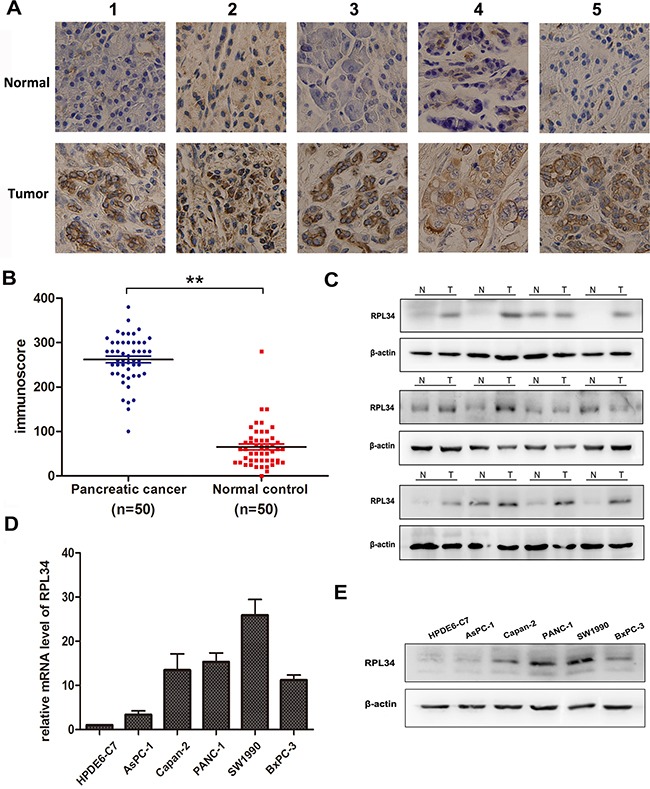
RPL34 is overexpressed in pancreatic cancer tissues and cell lines **A.** RPL34 expression in pancreatic tumor and matched normal pancreatic tissues was detected using IHC staining (40×). **B.** IHC staining of RPL34 was quantified by analyzing immunoscore as described in Materials and Methods. **P < 0.01 **C.** RPL34 expression in pancreatic tumor (T) and normal pancreatic tissues (N) was detected by western blot. β-actin was used as a loading control. **D.** RPL34 mRNA in pancreatic cancer cells was detected by qRT-PCR. **E.** RPL34 in human pancreatic cancer cells was detected by western blot. The normal pancreatic epithelial cell line HPDE6-C7 was used as a negative control and β-actin was used as loading control in D and E.

We used immunohistochemical staining to assess RPL34 expression in 50 pairs of PC and matched adjacent normal tissues. Strong RPL34 staining was detected in almost all PC specimens, while weak staining was observed in matched normal tissues (Figure 1A). The RPL34 immunoscore was significantly higher in PC tissues than matched adjacent normal tissues (Figure 1B). We then confirmed RPL34 expression in 12 pairs of these specimens using western blotting, the results of which were consistent with immunohistochemical staining of the tissues (Figure 1C).

To examine the role of RPL34 in PCs, we used western blotting and qRT-PCR to measure its expression in a panel of PC cell lines and the normal human pancreatic epithelial cell line HPDE6-C7. RPL34 mRNA levels were significantly higher in PC cells than that in normal HPDE6-C7 cells, and expression of RPL34 was highest in SW1990 and PANC-1 (Figure 1D). Consistent with the up-regulation of mRNA, immunoblotting analysis demonstrated that levels of RPL34 protein were also higher in PC cells than that in normal HPDE6-C7 cells, and were highest in SW1990 and PANC-1 cells (Figure 1E). Together, these results showed that RPL34 was up-regulated in PC cells and tissues.

To evaluate the correlation between RPL34 expression level and the clinical pathologic characteristics of these 50 PC patients, the median RPL34 level was set as the cut-off point for low and high expression. As shown in Table 1, RPL34 levels were closely correlated with p-AJCC stage (*P* = 0.016), lymph node metastasis (*P* = 0.005) and angiolymphatic invasion (*P* = 0.021) in PC patients, but were not significantly associated with age or differentiation grade. These data indicated that high levels of RPL34 predicted development of a worse PC.

**Table 1:** Clinical pathologic characteristics and RPL34 expression in 50 Pancreatic Cancers

Characteristics	NO. of patients	Expression of RPL34		*P*-value
		low^#^	high^#^	
**Overall**	50	25	25	
**Age**				
≥ 60	22	10	12	0.569
< 60	28	15	13	
**Gender**				
Male	32	15	17	0.556
Female	18	10	8	
**Tumor size**				
≥ 20 mm	34	14	20	0.069
< 20 mm	16	11	5	
**Differentiation**				
Well	6	3	3	0.912
Moderate	32	15	17	
Poor	12	7	5	
**Peripancreatic lymph**				
Negative	24	17	7	0.005**
Positive	26	8	18	
**pAJCC stage^##^**				
I	5	4	1	
IA	17	11	6	0.016*
IB	27	10	17	
II	1	0	1	
V	0	0	0	
**Perineural invasion**				
Negative	5	3	2	0.637
Positive	45	22	23	
**Angiolymphatic invasion**				
Negative	20	14	6	0.021*
Positive	30	11	19	

# Median RPL34 level is used as the cut-off.

## pAJCC pathologic tumor stage was determined according to the American Joint Committee on Cancer

(AJCC.2010)

* P < 0.05

** P < 0.01.

### Decreased methylation of the RPL34 promoter is correlated with the up-regulation of RPL34 in PCs

**Figure 2:**
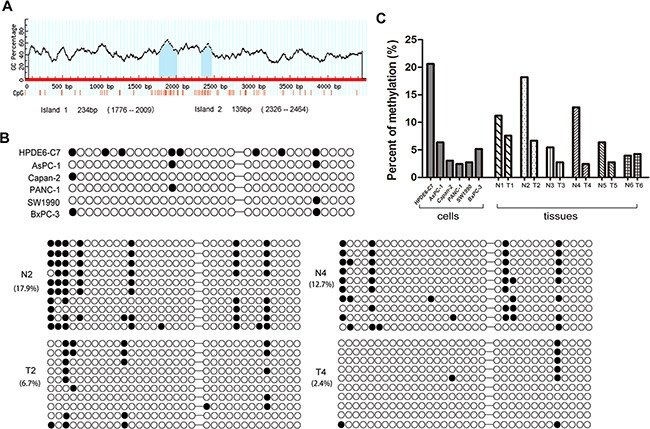
Methylation status of RPL34 promoter region in PC cells and tissues **A.** Position of CpG islands in the promoter transcriptional regulator region of RPL34. **B.** Methylation status of each of the 33 CpG sites in 6 pancreatic cancer cells and 2 pairs of PC tissues was detected by bisulfite sequencing PCR (BSP). Black circles represent methylated CpG sites; white circles represent unmethylated CpG sites. BSP cloning-based sequence analysis was performed and representative data for each group are shown. **C.** The methylation level of CpG regions were analyzed in 6 pairs of PC tissues.

There are two CpG islands, including 33 CpG sites, 2500 bp upstream of the RPL34 promoter transcriptional regulator region (TRR) (Figure 2A), suggesting that the methylation status of the RPL34 promoter may contribute to up-regulation of RPL34 in PCs. We collected human PC and pancreatic epithelial cells, and PC and corresponding normal tissues from 10 patients and performed bisulfite sequencing PCR to assess methylation status. We detected fewer methylated CpG sites in the PC cells or tissues with higher levels of RPL34 expression than in normal cells and corresponding normal tissues (Figure 2B and 2C). These results suggest that decreased methylation of the RPL34 promoter may contribute to the up-regulation of RPL34 in PCs.

We further treated HPDE6-C7 cells (higher-methylated) and PANC-1 and SW1990 cells (lower-methylated) by using demethylating agent 5-Aza-dC and/or the reversible histone deacetylase inhibitor TSA, as shown in Supplementary Figure S1, both mRNA and protein levels of RPL34 increased after 5-Aza-dC and TSA treatment in HPDE6-C7 cells, but no change was observed in PANC-1 and SW1990 cells. Taken together, These results strongly support our conclusion that RPL34 is regulated by the methylation of its promoter.

### Knockdown of RPL34 inhibits growth and proliferation of PC cells *in vitro*

**Figure 3:**
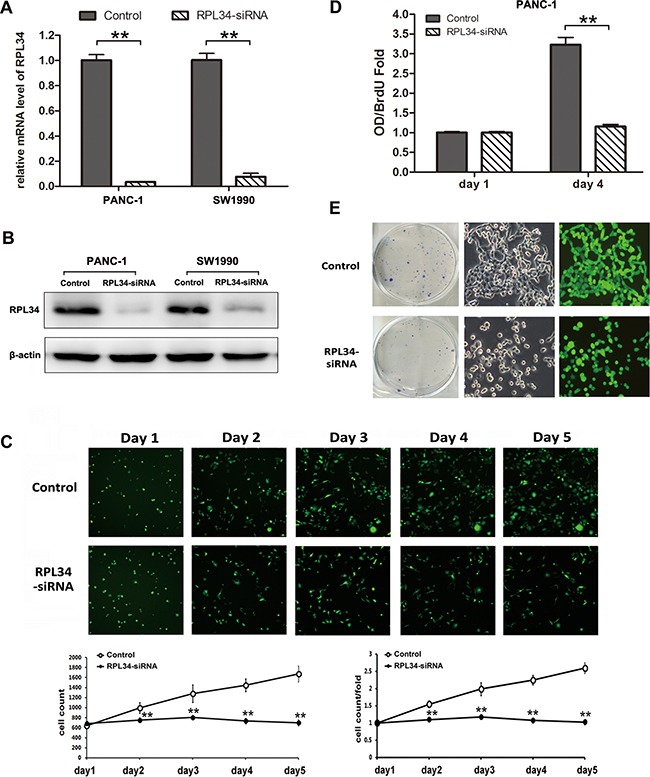
Knockdown of RPL34 inhibits cell growth and proliferation **A.** RPL34 mRNA level was assessed by quantitative RT-PCR in two PC cell lines. **P < 0.01. Control, cells infected with negative control lentivirus; RPL34-siRNA, cells infected with RPL34-siRNA lentivirus. **B.** PC cell lineRPL34 protein content was assessed by western blot. **C.** Cell growth was measured by multiparametric high-content screening (HCS) for five days in PANC-1 cells. **D.** DNA synthesis was analyzed by BrdU incorporation assay on the 1^st^ and 4^th^ days. Data are represented as mean ± SD.**P < 0.01. **E.** Colony formation was assessed by colony formation assay. Data presented represent three independent experiments (left). A single colony from each group was magnified (right) (40×).

To assess the role of RPL34 in regulating malignant PC phenotypes, we used lentivirus-delivered siRNA to knock down RPL34 expression in PANC-1 and SW1990 cell lines, both of which expressed high levels of RPL34. After 48 h, more than 85% cells were transduced with RPL34-siRNA, and qRT-PCR analysis indicated that RPL34 mRNA levels were significantly reduced in both cell lines (Figure 3A). Similar reductions in RPL34 protein level were detected by western blot in both cell lines (Figure 3B). Then high-content screening assay (HCS) were performed to monitor cell growth for 5 days. As shown in Figure 3C, silencing of RPL34 decreased total cell numbers and slowed the growth rate of PANC-1 cells. The BrdU incorporation DNA synthesis assay demonstrated that RPL34 siRNA significantly reduced proliferation of PANC-1 cells for days (Figure 3D). These results indicate that siRNA silencing of RPL34 expression significantly decreased DNA synthesis for 4 days, which resulted in slowed cell growth.

In order to assess the effect of RPL34 on PC cell tumorigenesis *in vitro* we studied colony formation of cells in which RPL34 was knocked down by siRNA. The number of colonies formed by RPL34 deficient PANC-1 cells (42.67±6.03) was significantly lower than the number formed by control cells (119.67±10.01, *P* < 0.01), and the morphology of RPL34 deficient PANC-1 cells also differed from control cells (Figure 3E). We obtained similar results in other cell lines, including SW1990 and BxPC-3, transduced with RPL34 siRNA (Supplementary Figure S4A and Figure S5A). We also confirmed overexpression of RPL34 moderately promoted cell proliferation and colony formation (Supplementary Figure S2A-D). Furthermore, we tested the efficacy of knocking down RPL34 on PANC-1 cell chemosensitivity to gemcitabine and 5-fluorouracil (5-Fu). As shown in Supplementary Figure S3, knockdown of RPL34 sensitized the tumor cells to gemcitabine and 5-Fu. Taken together, these results indicate that RPL34 is critical for the proliferation of PC cells and cell sensitivity to chemotherapies.

### Knockdown of RPL34 induces cell cycle arrest and apoptosis of PC cells

**Figure 4:**
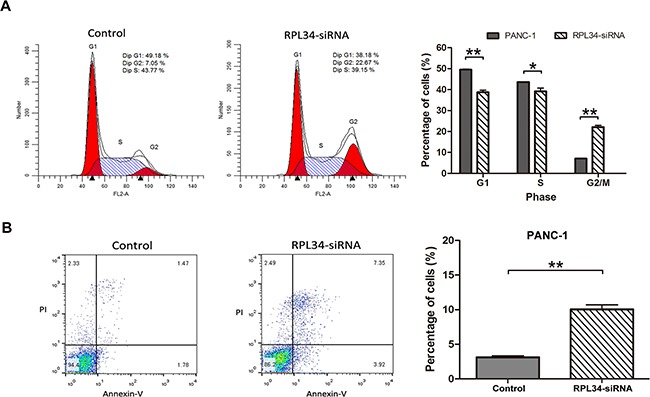
Knockdown of RPL34 induces cell cycle arrest and apoptosis **A.** Cell cycle distribution of NC and RPL34-siRNA PANC-1 cells was detected using PI staining, analyzed by flow cytometry, indicating the percentage of cells in the G0/G1, S and G2/M phases. Data presented represented the mean ± SD of three independent experiments.** *P* < 0.01. **B.** Apoptosis was assessed using Annexin-V/PI double staining, analyzed by flow cytometry. The cells in different stages of apoptosis/death were represented. The rate of apoptosis was represented as mean ± SD. *P < 0.05, **P < 0.01.

To assess whether RPL34 promotes proliferation of PC cells by regulating cell cycle progression or apoptosis, we used PI staining to measure cell cycle distribution and Annexin-V staining to assess apoptosis in RPL34 deficient and control PANC-1 cells. PANC-1 cells transduced with control siRNA had the following cell cycle distribution: G0/G1 49.18%, S 43.77%, G2/M 7.05%; siRNA RPL34 knockdown significantly reduced the fraction of cells in the G0/G1 and S phase, and significant increased the fraction in the G2/M phase, with the following cell cycle distribution: G0/G1 38.18%, S 39.15%, G2/M 22.67% (all *P* < 0.01, Figure 4A). We also confirmed overexpression of RPL34 increased the fraction of cells in the S phase, with no obvious change in G2/M phase in AsPC-1 cells (Supplementary Figure S2E). These data demonstrate cell cycle progression through G2/M phase was hindered in PANC-1 cells after RPL34 silencing. Furthermore, RPL34 knockdown significantly induced apoptosis in PANC-1 cells (RPL34-siRNA 10.04 ± 1.12% vs. control 3.13 ± 0.33%) (Figure 4B). Similar results were observed in SW1990 and BxPC-3 cells following RPL34 silencing by siRNA (Supplementary Figure S4B-C and Figure S5B-C). These results suggest that RPL34 enhances cell cycle progression and inhibits apoptosis in PC cells.

### Knockdown of RPL34 inhibits migration and invasion of PC cells

**Figure 5:**
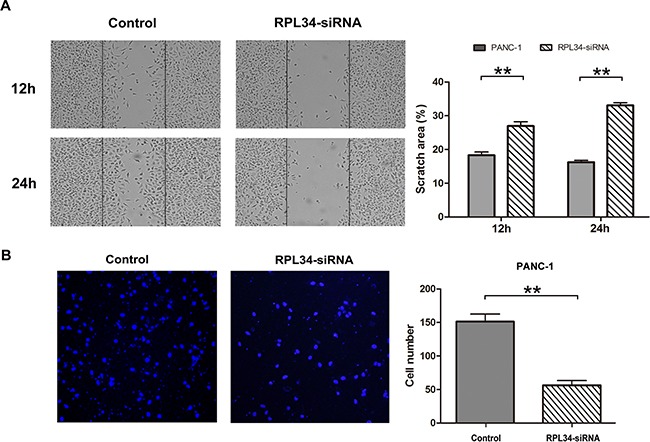
Knockdown of RPL34 inhibits cell migration and invasion **A.** Cell migration in NC and RPL34-siRNA PANC-1 cells was assessed by wound healing assay. The would closure fields were imaged at 12 and 24 h. **B.** Cell invasion was detected by transwell assay (40×). Data presented represents the mean ± SD of three independent experiments.**P < 0.01.

The alteration of the morphology of PC cells after RPL34 silencing (Figure 3E) led us to ask whether RPL34 influences cell migration and invasion. To test this hypothesis, wound-healing and transwell assays were performed to investigate the role of RPL34 in cell migration and invasion of PANC-1 cells. RPL34 knockdown substantially delayed wound closure of scratch gaps within 12 h and 24 h (Figure 5A). Transwell assays revealed PANC-1 cells with RPL34 silencing demonstrated reduced invasion ability, with significantly less cells invaded to the filter in comparison to control cells (Figure 5B). We further confirmed overexpression of RPL34 in AsPC-1 cells promoted cell migration (Supplementary Figure S2F). These data suggest that RPL34 might promote the progression of PC by accelerating cell migration and invasion.

### Knockdown of RPL34 suppresses pancreatic cancer cell growth and metastasis *in vivo*

**Figure 6:**
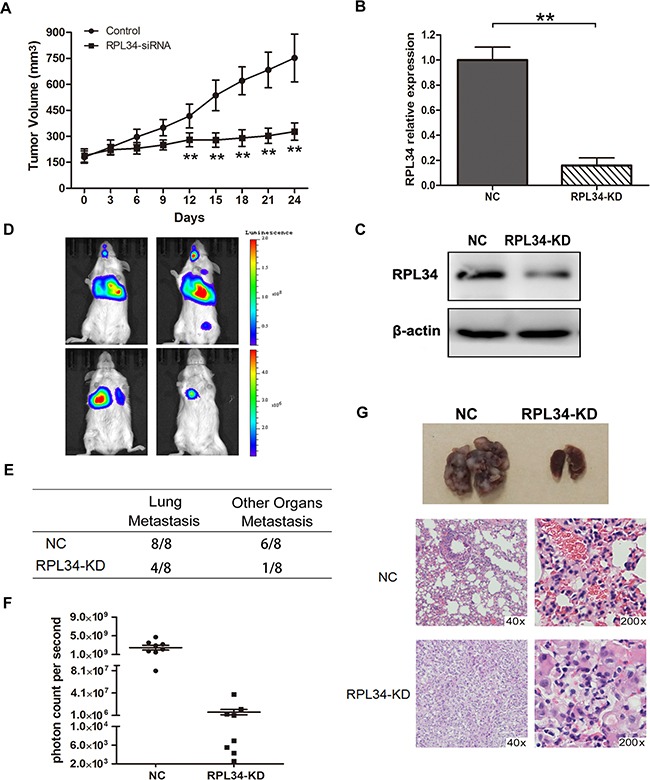
Knockdown of RPL34 in PANC-1 cells suppresses tumor growth and lung metastasis *in vivo* **A.** Xenograft models (n = 8) in nu/nu mice were generated with PANC-1 NC and RPL34-siRNA stably transfected cells. Tumor size was measured every other day for 24 days. Lung metastasis models (n = 8) in SCID mice were generated with luciferase- labeled PANC-1 cells transfected with shRNA of RPL34 (KD) or NC. RPL34 expression in luciferase-labeled PANC-1 cells transfected with shRNA of RPL34 (KD) or NC was assessed by **B.** qRT-PCR and **C.** Western blot. **D.** Luminescence was assessed in the 8 nude mice in each group 6 weeks after injection, and representative images for each group are shown. **E.** The incidence of metastases in the lung or other organs in the RPL34 and NC group. **F.** To quantify metastasis mass, the photon counts per second were recorded (***P < 0.001). **G.** The lungs of nude mice from each group were removed and lung metastases were evaluated by H&E staining.

To extend our *in vitro* observations *in vivo*, we generated PC mouse xenografts of PANC-1 cells stably transfected with lentivirus-based pGCSIL-GFP vector carrying RPL34-siRNA or negative control siRNA. 24 days after injection, the volume of RPL34-deficient xenograft tumors were significantly smaller than control xenograft tumors (Figure 6A).

To assess the effect of RPL34 on pancreatic tumor metastasis *in vivo*, we generated lung metastasis SCID mice models with PANC-1-luc-NC and PANC-1-luc-KD cells. RPL34 mRNA and protein levels were lower in transplanted PANC-1-luc-KD cells than PANC-1-luc-NC cells (Figure 6B and 6C). The location and growth of tumor xenografts *in vivo* was monitored by luciferase imaging once a week. As shown in Figure 6D, visible lung tumor metastases were observed in all the mice which received PANC-1-luc-NC cells (n = 8), and 6 mice developed metastases in other organs, including the brain, liver or bone; however, there was a lower incidence of lung or other organs metastases in RPL34KD group than that in control group, in which lung metastases were detected in three mice and other organ metastases were detected in one mouse that received PANC-1-luc-KD cells (n = 8) (Figure 6E). Moreover, the bioluminescence of PANC-1-luc-NC metastases was stronger than that of PANC-1-luc-KD metastases (Figure 6F). Histological analysis of the lungs revealed fewer and smaller metastatic tumors in mice that received PANC-1-luc-KD cells than mice that received PANC-1-luc-NC cells (Figure 6G). Taken together, these data demonstrate that RPL34 silencing could effectively reduce tumor growth and metastasis of PC *in vivo*.

### RPL34 may promote the tumorigenesis of PCs through MAPK and p53 signaling pathways

**Figure 7:**
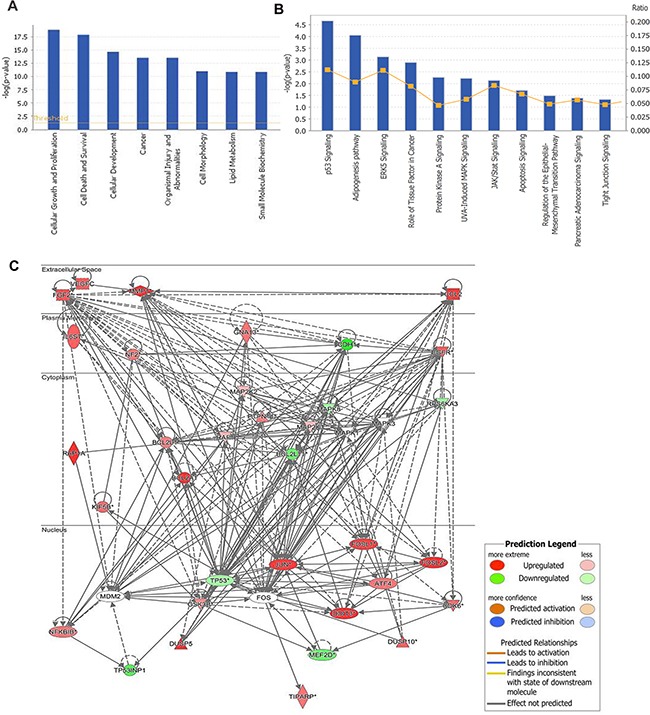
Gene expression profiling identified an association between changes in RPL34 expression and MAPK and p53 signaling **A.** Diseases and functions analysis classified genes enrichment after RPL34 silencing. **B.** Annotated classical pathways analysis indicated genes enriched after RPL34 silencing. **C.** Knowledge-based interaction network of MAPK and P53 targets after RPL34 silencing in PANC-1 cells. The network was built based on the MAPK interactome in the Ingenuity IPA database overlaid with microarray data from RPL34-KD cells with a 2-fold change cut-off. The intensity of the node color indicates the degree of up- (red) or down- (green) regulation following RPL34 knockdown in PANC-1 cells.

To elucidate the molecular mechanisms by which RPL34 promotes malignancy of PC cells, microarray analysis was performed to compare gene expression in cells transduced with PANC-1 NC-siRNA and RPL34-siRNA. The data revealed that expression of 557 genes differed by at least 2-fold (*P* < 0.05). Further disease and function analysis revealed that the following particular gene sets that were significantly enriched, including those involved in cellular growth and proliferation, cell death and survival, cellular development, cancer, organism injury and abnormalities (Figure 7A). Next, classical pathway analysis revealed that the MAPK and P53 signaling pathways were altered following RPL34 silencing (Figure 7B). To further confirm the contribution of MAPK signaling in RPL34-induced malignancy, we analyzed the knowledge-based interactome surrounding the regulation of MAPK signaling using Ingenuity Pathway Analysis (IPA) and overlaid our microarray data with a 2-fold change cut-off. Several genes that have been reported to be involved in the process of tumorigenesis were differentially regulated by RPL34, including CCL2, ATF4, MMP1, Bcl-2, EGFR, CDK6, MDM2, FOSL2 and JUN (Figure 7C). These data implicate the MAPK signal pathway in the mechanism by which RPL34 promotes PC tumorigenesis.

## DISCUSSION

In the past decade, several ribosomal proteins have been found to be involved in the tumorigenesis and progression of various cancers. RPL34 is a rarely studied ribosomal protein and most previous studies mainly focused on its expression and functions in mosquitoes and plants [18, 19]. Recently, two reports revealed RPL34 promoted cell proliferation in non-small cell lung and gastric cancers [14, 15]; however, the roles for RPL34 in cell malignances and the mechanisms of its dysregulation remain unexplored in PCs.

In this study, we found that RPL34 expression was upregulated in PC tissues and positively correlated with pAJCC stage, lymphatic metastasis and angiolymphatic invasion in PC patients, which indicates RPL34 might play a crucial role in tumor development of PCs. Aberrant promoter methylation is usually responsible for the dysregulation of ribosomal protein expression in cancer tissues. For example, RPS6 expression is suppressed by promoter hypermethylation in CRC tissues [20], and PRMT5 acts as an oncoprotein by increasing methylation of RPS10 [21]. We analyzed the CpG sites and methylation levels of the RPL34 promoter TRR in PC cells and tissues, and corresponding normal cells and tissues. Our results demonstrated the CpG methylation status was inversely correlated with RPL34 expression, suggesting that demethylation of RPL34 promoter induces the overexpression of RPL34 in PCs.

Furthermore, using a lentivirus carrying RPL34 siRNA, we confirmed that silencing of RPL34 effectively inhibited the growth, proliferation, migration and invasion of PC both *in vitro* and *in vivo*. PC cell growth rate and colony formation was significantly lower following RPL34 silencing, RPL34 silencing caused both cell cycle arrest at G2/M phase and apoptosis. moreover, mouse xenografts of RPL34-siRNA cells grew more slowly than those of wild-type cells, producing smaller tumors. Intriguingly, RPL34 silencing altered cellular morphology, and reduced the migration and invasion of PC cells *in vitro* and *in vivo*. These results strongly suggest that RPL34 plays an oncogenic role in cell growth, proliferation and migration in PC.

To further explore the mechanisms underlying RPL34-mediated promotion of PC tumorigenesis, we performed microarray analysis of PANC-1-NC and PANC-1-RPL34-siRNA cells. Gene expression profiling revealed that RPL34 silencing enriched expression of genes involved in cellular growth and proliferation, cell death and survival, and cellular development and cancer, particularly MAPK and P53 signaling. The MAPK cascade is a critical pathway for human cancer cell proliferation, differentiation, migration, immune evasion and drug resistance, and aberrant activation of MAPK signaling is a frequent event in tumorigenesis, progression and metastasis of most tumors [22–25]. Further analysis of the knowledge-based interactome surrounding the regulation of MAPK via IPA analysis demonstrated that CCL2, ATF4, MMP1, EGFR, CXCL1 FOSL2, JUN and p53, which have been implicated in the proliferation and metastasis of various cancers [26–30], were found to be differentially regulated by RPL34 (> 2-fold, *P* < 0.05). P53 is a major tumor suppressor, it may also act as a downstream effector of MAPK, which contributes to the enhancement of cell cycle progression and proliferation by RPL34. Importantly, p53 usually acts as a central regulatory hub among many ribosome biogenesis regulators, and it has been well established that the MDM2-p53 feedback loop is regulated by ribosomal protein-mediated p53 activity like RPL4, RPS14 and RPS7 [31]. additionally, RPL34 could mediate the upstream factors (Foxo families) or downstream proteins (mTOR and AMPK) of p53 signaling pathway by a p53 independent manner when p53 is mutant or inactivated, like some reports found p53 mutants also play an effective role in inhibiting autophage [32]. Our findings suggest that RPL34 might be a novel regulator of the MDM2-p53 loop in PCs. However, the key molecules or pathways responsible for the malignant functions of RPL34 require further exploration.

Taken together, we found aberrant expression and identified novel functions of RPL34 in PC, and explored the molecular mechanisms underlying the tumorigenic role of RPL34 in PC. Our findings suggest that RPL34 is a potential biomarker for the diagnosis, prognosis and development of targeted therapy for PC.

## MATERIALS AND METHODS

### Patients and tissue samples

Fifty patients diagnosed with PC were enrolled at the Department of Hepatobiliary and Pancreas Surgery, the First Hospital of Jilin University between January 2011 and December 2013. The clinical and pathologic characteristics of the patient cohort are summarized in Table 1. All fresh tissue samples were paraffin-embedded, or snap-frozen immediately after resection and stored in liquid nitrogen. Informed consent was obtained form each patient and the protocols were approved by the Ethics Committee of the First Hospital of Jilin University.

### Immunohistochemistry analysis

The paraffin sections were deparaffinized, rehydrated in deionized water, and then exposed to 3% H_2_O_2_ and 1% BSA, followed by incubation with anti-RPL34 (1:50) antibody overnight and secondary antibody (RTU Vectastain kit, Vector Laboratories, Burlingame, CA) [16]. Sections were stained by DAB and hematoxylin. Images were acquired using an Olympus BX41 microscope. Semi-quantitative evaluation of IHC staining of RPL34 was carried out using immunoscore based on both the percentage of stained cells and staining intensity, as previously described [16, 17]. The intensity score was defined as follows: 0, no appreciable staining; 1, weak intensity; 2, moderate intensity; 3, strong intensity; 4, very strong intensity. The fraction score was based on the proportion of positively stained cells (0–100%). The mean of the immunoscores from ten microscopic high power fields was recorded.

### Cell culture and stably transfected cell lines

Human normal pancreatic epithelial cells HPDE6-C7 and PC cell lines PANC-1, SW1990, BxPC-3, Capan-2, AsPC-1 were obtained from the Type Culture Collection of the Chinese Academy of Sciences (Shanghai, China) and cultured in DMEM medium (Hyclone) supplemented with 10% fetal bovine serum (GIBCO) at 37°C in a humidified atmosphere containing 5% CO_2_. Stable PANC-1-luc-NC and PANC-1-luc-KD cell lines were established using lentivirus pGC-FU-LUC-IRES-Puromycin carrying control or RPL34-shRNA sequences. Cells were selected by culture in puromycin (2.5 μg/ml) and were continually passaged at low density to allow for election of subclones with acquired puromycin resistance.

### Lentivirus vector construction and transduction

Double-stranded RPL34-targeted siRNA sequences (5′- CCTAAAGTTCTTATGAGAT-3′) were synthesized and cloned into the lentivirus-based pGCSIL-GFP vector with AgeI/EcoRI sites by GeneChem Corporation (Shanghai, China). The negative control lentiviral vector (5′-TTCTCCGAACGTGTCACGT-3′) was generated, which shared no homology with any known human genes. For lentivirus transduction, cells were seeded in six-well plates until cell confluence reached 70%, then lentivirus was added to achieve the multiplicity of infection of 1. After 72 h, cells were observed under a fluorescence microscope and harvested for the following experiments.

### Bisulfite sequencing PCR (BSP) of the *RPL34 *gene

The primers used to detect methylation of the CpG islands were designed to specifically amplify bisulfite-converted DNA of RPL34 promoter transcriptional regulator region (TRR). The primers were RPL34-1F: 5’-AAGTGTTTATGATAA(C/T)GAAAGAAGTTG-3’; RPL34-1R: 5’-AACAACATCCATACCTACAAACAAC-3’; RPL34-2F: 5’-AGGGATTTTG TTGTATTTTAAGTGTAG-3’; RPL34-2R: 5’-AAAAACCATTATTCTATTTACATCT CC-3’. Aliquots of the bisulfite-modified DNA from PC cells or tissues were subjected to PCR analysis. The reaction was preheated at 95°C for 5 min, and amplified using a touch-down PCR program: 10 cycles of 95°C for 30 s, 60°C for 45 s (with touch down 1°C per cycle), and 72°C for 45 s; 30 cycles of 95°C for 30 s, 50°C for 45 s, and 72°C for 45 s, and finally 60°C for 30 min. The PCR products were cloned into the pMD 18-T Vector (TaKaRa), then 10 clones from each sample were randomly selected for DNA sequencing by Comate Bioscience (Jilin, China).

### Cell growth and proliferation assays

Multiparametric high-content screening (HCS) was used to determine cell growth. Briefly, cells were seeded in 96-well plates (1,000 cells/well) and continuously cultured for 5 days. Plates were processed with the ArrayScan™ HCS software (Cellomics Inc. Halethorpe, MD, USA) each day. The system includes a computerized, automated fluorescence-imaging microscope that automatically identifies stained cells and reports the intensity and distribution of fluorescence in each cell. Images were acquired for each fluorescence channel (20 ×). To measure proliferation, DNA synthesis was assessed by BrdU incorporation assay using a Brdu kit (Roche) following the manufacturer's instructions, with the cells pulsed with Brdu for 12h at each time point.

### Cell migration and invasion assay

For cell migration assay, cells were seeded in 6-well plates (1 x 10^5^ cells/well) and a scratch wound was made by scraping confluent cells with sterile pipette tips, and images were captured 12 h or 24 h later. For invasion assay, cells in 200 μl serum-free 1640 medium were seeded in the upper insert of 6.5 mm transwell chambers with 8 μm pores (Costar, Cambridge, MA, USA) pre-coated with matrigel (1:4, BD), then 500 μl complete medium was added into the lower chambers. After 24 h, medium was removed and the upper surface of each membrane was cleaned with cotton swab. The cells adhering to the insert surface were stained with DAPI and images were acquired using Olympus BX41 microscopy.

### Establishment of pancreatic cancer xenograft model in nude or SCID mice

Animal experiments were performed following the protocols approved by Institutional Animal Care and Use Committees of Jilin University. Nude (BALB/C-nu/nu) mice and SCID (severe combined immunodeficient) mice were obtained from Vital River Laboratory Animal Technology (Beijing, China). 5×10^6^ PANC-1 cells stably transduced with lentivirus-based pGCSIL-GFP vector carrying negative control and RPL34-siRNA were suspended in HBSS and injected subcutaneously into the flank region of 6-week-old female athymic nude (BALB/C-nu/nu) mice (Vital River Laboratory Animal Technology, Beijing, China). The tumors were monitored and grown to an average volume of 200 mm^3^. Tumor size was assessed by caliper every other day and tumor volumes were calculated after 24 days using the formula: V = 4/3 × π (length/2 × (width/2)^2^).

To establish a metastasis model, SCID mice were injected with 1× 10^6^ viable PANC-1-luc-NC and PANC-1-luc-KD cells via tail veins. Successful injection was confirmed by immediate luciferase imaging. For luciferase imaging, mice were anesthetized with isoflurane and intraperitoneally injected with luciferin (25 mg/ml in 0.1 ml PBS). Animals were imaged 15 min after injection using an IVIS LuminaXR system (Caliper, Hopkinton, MA, USA) once a week for a total of six weeks.

### Statistical analysis

All Statistical analyses were performed using GraphPad Prism software (San Diego, CA, USA). Data are presented as the mean ± standard error, and the statistical significance between two groups was assessed using two-tailed unpaired Student's t-test. The association between RPL34 expression and patients' clinical-pathologic characteristics was compared using Fisher's exact test. Data was considered statistically significant at *P* < 0.05.

## SUPPLEMENTARY MATERIALS AND METHODS


