# Supplementary material for: Ribosomal protein L34 promotes the proliferation, invasion and metastasis of pancreatic cancer cells
Source: Oncotarget. 2016 Nov 10;7(51):85259–72. doi: 10.18632/oncotarget.13269 (PMC5356734; doi:10.18632/oncotarget.13269)
Supplement: Supplementary file 1 [file oncotarget-07-85259-s001.pdf]

Ribosomal protein L34 promotes the proliferation, invasion and metastasis of pancreatic cancer cells

SUPPLEMENTARY MATERIALS AND METHODS

Cell proliferation assay (SRB assay)

Cells were seeded in 96-well plates at 8,000 per well and cultured overnight. Cells were treated with gemcitabine or 5-fluorouracil for 48 h, or collect cells at different time point without treatment. The survival cell fraction was determined using the sulforhodamine B (SRB) assay as described (1). The percentage of cell viability was calculated by using the equation: % cell viability = (At/Ac) × 100%, in which At and Ac represent the absorbance in treated and control cultures respectively.

RNA isolation and quantitative RT-PCR

Total RNAs were extracted from cell cultures and snap-frozen tissues by using Trizol (Invitrogen) according to the manufacture's protocols. cDNA was synthesized from 4μg of total RNA with SuperScript III First-Strand Synthesis System (Invitrogen). Aliquots of reaction mixture were used for real-time PCR amplification. The primer for RPL34 were: sense: 5'-GCACA CATGG AACCA CCATA G-5' and antisense: 5'-GTTTG ACATA CCGAC GTAGG C-3'. All PCR experiments were performed in triplicate. GAPDH expression levels were used as control for data normalization. The relative expression of RPL34 was normalized to GAPDH using the $2^{-\Delta\Delta CT}$ method.

Western blot assay

Total proteins were extracted from cells or tissues by using cell lysis buffer with protease inhibitor cocktail (Thermo Scientific). Equal amount of proteins from each preparation were subjected to western blot according to standard protocols. After probing with primary antibody for RPL34 (Abcam, ab129394) and secondary antibodies, the membranes were developed using an ECL plus chemiluminescence kit (Beyotime, Shanghai, China).

Colony formation assay

Cells were trypsinized to a single cell suspension and seeded in 6-well plates (500/well), then cultured for 10 days and medium was replaced every three days. The colonies containing over 50 cells were counted after staining with crystal violet (0.1% in 20% methanol).

Cell cycle and apoptosis assay

For cell cycle analysis, cells were synchronized by 48 h serum starvation and released into cell cycle by the addition of complete medium containing 10% FBS. After incubation for 24 h, the cells were harvested, fixed with 70% ethanol, and stained with propidium iodide (PI), which was analyzed by flow cytometry. To assess apoptosis, 1×10^6 cells were suspended in binding buffer, stained with Annexin V and PI, incubated for 15 min in the dark and subjected to flow cytometry analysis. All data were acquired by FACScan (BD Biosciences) and analyzed using FlowJo software (Tree Star Inc. Ashland, OR, USA).

REFERENCES

1. Vichai, V., and Kirtikara, K. (2006) Sulforhodamine B colorimetric assay for cytotoxicity screening. Nat Protoc 1, 1112-1116.

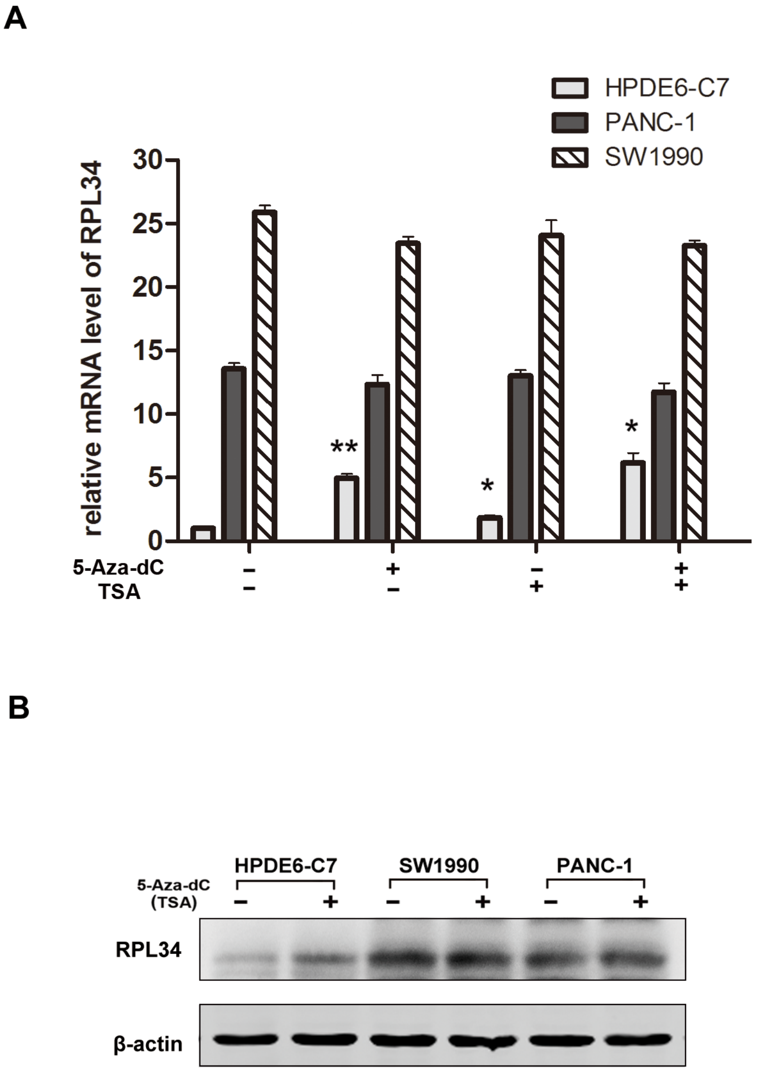

Supplementary Figure S1: Demethylation of RPL34 promoter increases the expression of RPL34. A. RPL34 mRNA and **B.** protein levels in normal pancreatic epithelial cells (HPDE6-C7, higher-methylated) and pancreatic cancer cells (PANC-1, SW1990, lower-methylated) were analyzed by real-time PCR (A) and western blot (B) after treatment with 5-Aza-dC, TSA, respectively or combination. β-actin was used as loading control. *P < 0.05. **P < 0.01 vs. negative control (NC).

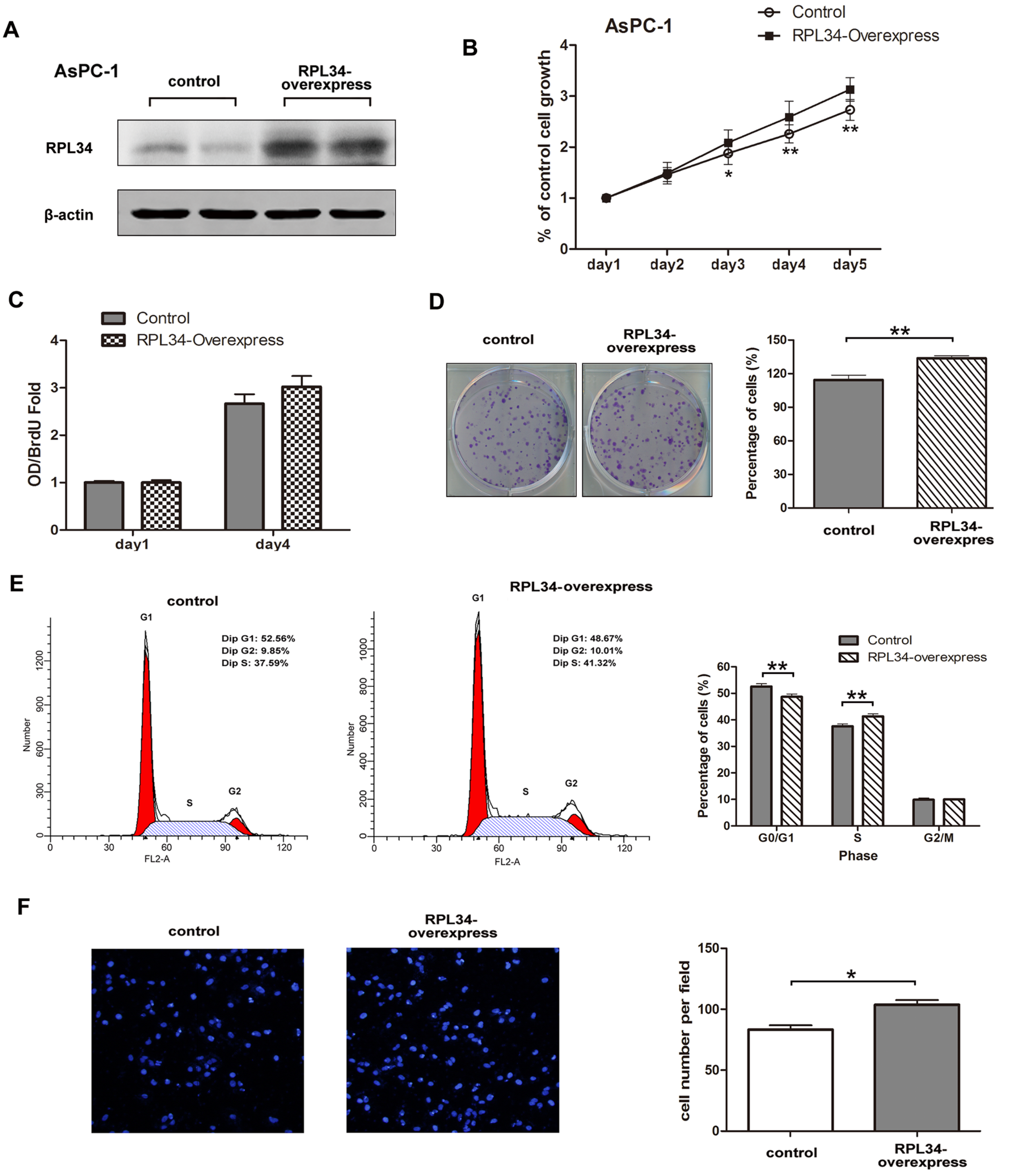

Supplementary Figure S2: Overexpression of RPL34 moderately promote cell proliferation and metastasis. PANC-1 cells was transfected with pcDNA3.1-RPL34 plasmid or vector control, **A.** RPL34 mRNA level was assessed by western blot. **B.** Cell growth was analyzed by SRB assay. **C.** DNA synthesis was analyzed by BrdU incorporation assay on the 1st and 4th days. **D.** Colony formation was assessed by colony formation assay. (Right), quantitative analysis of cell clones. **E.** Cell cycle distribution of NC and RPL34-overexpression cells was detected by using PI staining and analyzed by flow cytometry. (Right), quantitative analysis of cell proportion in different phase. **F.** Cell metastasis was detected by transwell assay (40×).(Right), quantitative analysis of transwelled cells. Data presented represents the mean ± SD of three independent experiments. *P < 0.05. **P < 0.01 vs. NC.

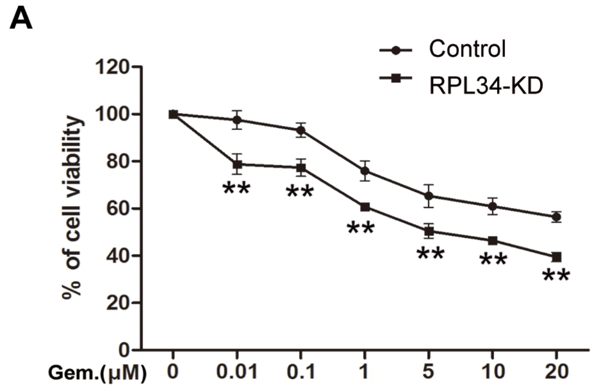

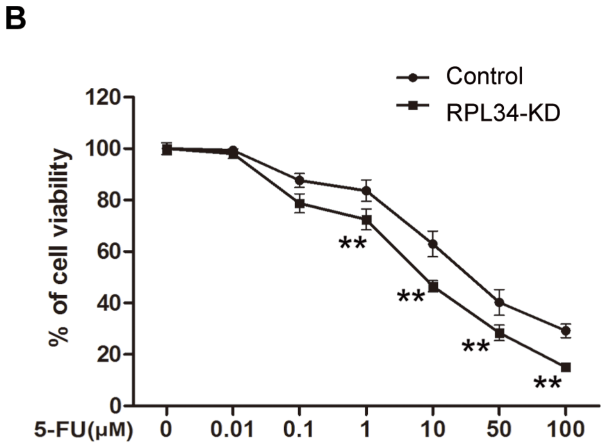

Supplementary Figure S3: Knockdown of RPL34 sensitizes PANC-1 cells to chemo-treatments. PANC-1 cells was treated with gemcitabine **A.** or 5-fluorouracil **B.** for 48 h at different concentrations respectively. The survival cell fraction was determined using the SRB assay. Data presented represented the mean ± SD of three independent experiments performed in triplicate. *P < 0.05. **P < 0.01 vs. NC.

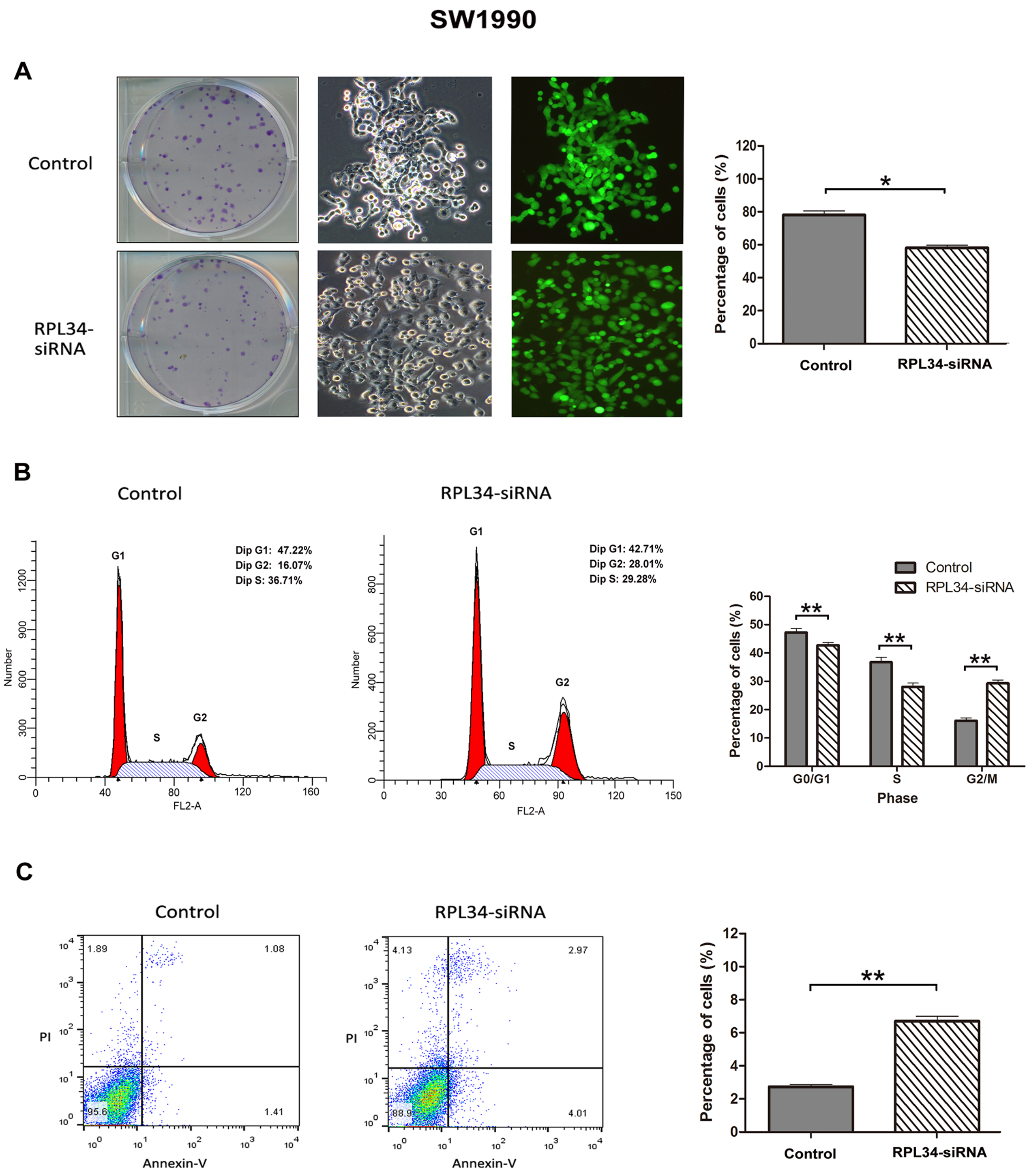

Supplementary Figure S4: Knockdown of RPL34 inhibits cell growth and induces cell apoptosis in SW1990 cells. Control, cells infected with negative control lentivirus; RPL34-siRNA, cells infected with RPL34-siRNA lentivirus. **A.** Colony formation was assessed by colony formation assay, a single colony from each group was magnified (40×). (Right), quantitative analysis of cell clones. **B.** Cell cycle distribution was detected by using PI staining and analyzed by flow cytometry. (Right), quantitative analysis of cell proportion in different phase. **C.** Cell apoptosis was assessed using Annexin-V/PI double staining and analyzed by flow cytometry. (Right), quantitative analysis of apoptotic cells (including early and late stages). Data presented represents the mean ± SD of three independent experiments. *$P < 0.05$. **$P < 0.01$ vs. NC.

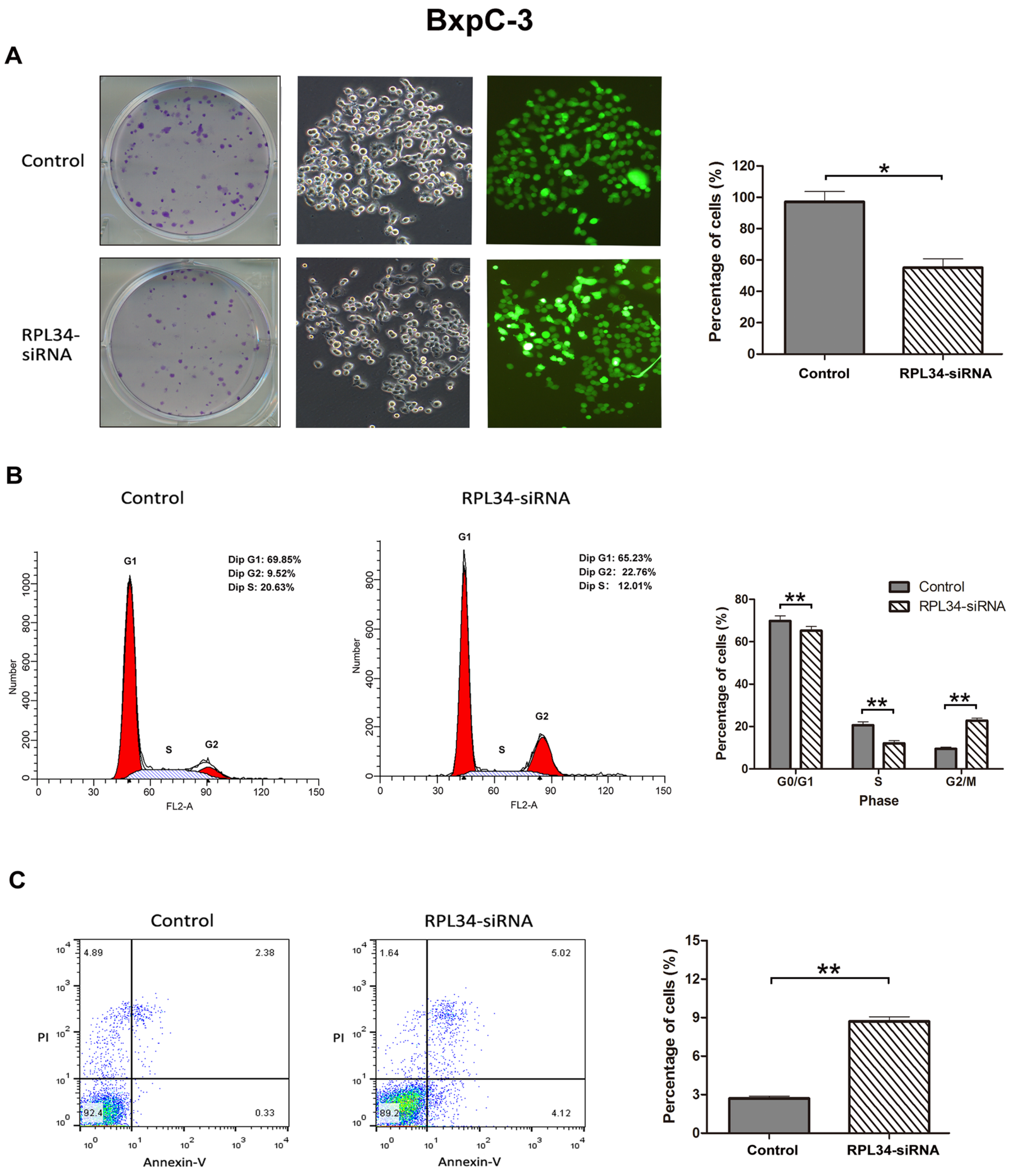

Supplementary Figure S5: Knockdown of RPL34 inhibits cell growth and induces cell apoptosis in BxPC-3 cells. Control, cells infected with negative control lentivirus; RPL34-siRNA, cells infected with RPL34-siRNA lentivirus. **A.** Colony formation was assessed by colony formation assay, a single colony from each group was magnified (40×). (Right), quantitative analysis of cell clones. **B.** Cell cycle distribution was detected by using PI staining and analyzed by flow cytometry. (Right), quantitative analysis of cell proportion in different phase. **C.** Cell apoptosis was assessed using Annexin-V/PI double staining and analyzed by flow cytometry. (Right), quantitative analysis of apoptotic cells (including early and late stages). Data presented represents the mean ± SD of three independent experiments. *P < 0.05. **P < 0.01 vs. NC.
